# The First Records of Canine Babesiosis in Dogs from *Dermacentor reticulatus*—Free Zone in Poland

**DOI:** 10.3390/pathogens11111329

**Published:** 2022-11-11

**Authors:** Olga Pawełczyk, Damian Kotela, Marek Asman, Joanna Witecka, Peter Wilhelmsson, Paulina Bubel, Krzysztof Solarz

**Affiliations:** 1 Department of Parasitology, Faculty of Pharmaceutical Sciences in Sosnowiec, Medical University of Silesia, 40-055 Katowice, Poland; 2 Veterinary Clinic for Small Animals “DAWET”, 42-400 Zawiercie, Poland; 3 Department of Medical and Molecular Biology, Faculty of Medical Sciences in Zabrze, Medical University of Silesia, 40-055 Katowice, Poland; 4 Department of Biomedical and Clinical Sciences, Division of Inflammation and Infection, Linköping University, 581 83 Linköping, Sweden; 5 Department of Clinical Microbiology, Region Jönköping County, 556 52 Jönköping, Sweden

**Keywords:** *Babesia canis*, canine babesiosis, tick-borne pathogens, tick-borne diseases, *Dermacentor reticulatus*, ticks, Poland, Central Europe

## Abstract

Tick-borne microorganisms belong to important etiological agents of many infectious diseases affecting humans and animals. Among them, there are haemoprotozoans of the *Babesia* genus, which infect erythrocytes of a host and may cause many clinical symptoms. Canine babesiosis is an emerging tick-borne disease in Southern and Central Europe. In this study, we report two cases of symptomatic canine babesiosis caused by *Babesia canis* in domestic dogs from the Silesian Voivodeship, Poland, as well as the presence of *Dermacentor reticulatus* ticks detected on one of the *Babesia*-infected dogs (*D*. *reticulatus*-free zone). The molecular analysis confirmed the presence of *Babesia canis* in the dogs’ blood, and the sequencing analysis showed that the obtained sequence is 100% identical to the sequence of *Babesia canis* isolate 3469 (sequence ID: KX712122.1). Our findings should raise awareness of *B. canis* infection among dog owners and veterinarians in the region where *B. canis* was not previously reported in residential, non-traveling dogs, as well as ensuring that adequate diagnostic methods are available.

## 1. Introduction

Tick-borne microorganisms are significant etiological agents of many infectious diseases affecting humans and animals worldwide. Some of the tick-borne pathogens (TBP) are protozoans of the genus *Babesia*, parasites that infect erythrocytes of the host and cause many clinical manifestations [1,2,3].

In Europe, four species of *Babesia* are recognized to be pathogenic for dogs, including the large-sized (3.0–5.0 µm)-*Babesia canis* and *B. vogeli*, as well as the small-sized (1.5–2.5 µm)-*B. gibsoni* and *B. microti*-like (*B. vulpes*/*Theileria annae/Babesia* “Spanish dog isolate”) [4,5]. Until now, only two of them-*B. canis* and *B. gibsoni* have been reported in Poland [6,7,8,9].

Canine babesiosis is an emerging tick-borne disease affecting companion animals in countries of Southern and Central Europe, e.g., England, France, Portugal, Hungary, Croatia [10,11], Germany [12], Austria [13], Slovakia [14,15], as well as Poland [16,17]. Dog infections caused by European strains of *B. canis* may exhibit mild to severe symptoms. The clinical manifestations of acute clinical symptoms include lethargy, weakness, apathy, elevated temperature, dehydration, anorexia, icterus, splenomegaly, color change in mucous membranes and urine, hemoglobinuria, renal failure, vomiting, diarrhea, and tachycardia. A complete blood count usually presents many abnormalities, such as hemolytic anemia, leucopenia, neutropenia, thrombocytopenia, and lymphopenia [11,18]. *Babesia* infection is diagnosed by microscopic examination of May-Grünwald-Giemsa or Diff-Quick stained peripheral blood smears, with the finding of large pyriform parasites within the infected erythrocytes. Furthermore, polymerase chain reaction (PCR) analysis is a significant diagnostic tool for confirmation of the presence of the *Babesia* subspecies in dogs [19].

Different species of *Babesia* protozoans are transmitted to domestic and wild animals, as well as humans, mainly through tick bites. The occurrence of *B. canis* in dogs is closely related to the geographical range of the ornate dog tick-*Dermacentor reticulatus* (Fabricius, 1794), which is the main vector of this haemoprotozoan species [20,21]. In Poland, *D. reticulatus* commonly occurs in Eastern, Western and Central regions, and a similar pattern of canine babesiosis cases was recorded in this country, especially in Eastern and Central parts [22]. The occurrence of canine babesiosis reflects the seasonal activity of adult *D. reticulatus* ticks in this country, with two peaks of activity, the first in March-April (the spring peak of activity) and the second in September-October (the autumn peak of activity) [22,23]. Due to scientific reports, there is a confirmed *D. reticulatus*-free zone in Poland (so-called gap zone) which spreads from West Pomerania and Pomerania Voivodeships in Northern Poland to Opole, Silesia, Lesser Poland, and Subcarpatia Voivodeships in Southern Poland [24].

Our findings indicate that *B. canis* infection in domestic dogs, as well as *D. reticulatus,* is present in the previously mentioned *D. reticulatus*-free zone in Poland. Such information gives new light on the current scientific knowledge in this field and is especially valuable to veterinarians as well as dog owners.

## 2. Materials and Method

### 2.1. Dogs-Clinical Examination

Veterinary diagnostic procedures of an English Cocker Spaniel male (Dog 1) from Zawiercie (Silesia, Poland) included a detailed anamnesis, as well as a clinical examination with complete blood count (CBC) (automatic hematology analyzer Mindray BC-30 Vet, Stamar, Dabrowa Gornicza, Poland) and biochemical blood analyses (biochemical analyzer VETSCAN VS2, ZOETIS, Warsaw, Poland). The thin blood smears were prepared from the EDTA-treated blood samples taken from the cephalic vein and a Diff-Quik staining method (Hemavet Kit, KOLCHEM, Lodz, Poland). Blood smears allowed microscopic evaluation of *Babesia* protozoan’s size and shape in order to classify them as large or small forms (Nikon ECLIPSE E 200, Tokio, Japan).

A clinical examination of a Labrador Retriever female (Dog 2) from Zawiercie (Silesia, Poland) included an X-ray of the abdomen, and similarly as in the first case, an anamnesis, a complete blood count, a biochemical blood analysis, and a blood smear examination.

### 2.2. Blood and Ticks Analyses

Blood samples used in our study consisted of surplus material, which was collected for routine diagnosis from dogs brought to the veterinary clinic. Therefore, no formal ethical approval was needed. In order to use the surplus material for additional diagnostic tests, oral consent from the dog owners was obtained. Genomic DNA was extracted from 200 µL of EDTA-blood samples using a commercial Quick Blood DNA Purification Kit (EURx, Gdańsk, Poland) according to the manufacturer’s protocol. The concentration of DNA was measured spectrophotometrically at 260/280 wavelength in the Nanospectophotometer PEARL (Implen, Munich, Germany). DNA extracts were stored at −20 °C until use. *Babesia* spp. were detected in dogs’ blood by PCR method. To detect this protozoan, a pair of primers specific to the *18S* rRNA gene was used [25]. Then, the amplification product was separated electrophoretically in 2% ethidium bromide-stained gel and visualized under ultraviolet light. The PCR product was then isolated from the gel with the use of an Agarose-Out Kit (EURx, Gdańsk, Poland) according to the manufacturer protocol. Then, the samples were sequenced (Genomed, Warsaw, Poland), and the obtained sequences were compared with the sequences found in the GenBank database.

Two ticks were removed from Dog 1 by tick-tweezers during the clinical exam at the veterinary clinic. Both of them were placed separately in marked plastic tubes with 70% ethanol and transported to the Department of Parasitology (Medical University of Silesia in Katowice, Poland) in order to identify species and life stages of ticks, as well as molecular analysis. Ticks were identified under the stereomicroscope (Zeiss Stemi 2000C, Warsaw, Poland) using the identification keys [26,27]. Then, DNA was isolated from two ticks using the ammonium hydroxide method, according to previously described protocols [28], and the concentration of DNA was measured spectrophotometrically by the Nanospectrophometer PEARL (Implen, Munich, Germany). To detect *Babesia* spp. in ticks, a pair of primers specific to the *18S* rRNA gene was used [25]. In turn, to detect *A. phagocytophilum*, *B. burgdorferi* s.l., *Rickettsia* spp., and *Bartonella* spp., the primers specific to the *16S* rRNA gene, *flagellin* gene, *gltA* gene, and *rpoB* gene were used respectively [29,30,31,32]. The amplification products were separated electrophoretically in 2% ethidium bromide-stained gels and visualized under ultraviolet light.

## 3. Results

### 3.1. Dog 1-Clinical Examination and Molecular Analysis

In March 2021, a 2.5-years old male English Cocker Spaniel from Zawiercie (Silesia, Poland) was presented to the veterinary clinic for many clinical symptoms, such as weakness, lethargy, appetite disorder, and others (Table 1). During the examination, two engorged ticks were detected and removed from dogs’ skin. The medical history of this dog revealed no travel history in the last 2 months before the symptoms occurred. It had no anti-tick prophylaxis regardless of the fact that it was probably infected with canine babesiosis before, as a one-year-old puppy (information from the owners, Dog 1 was not a patient of this veterinary clinic before).

The complete blood count of the dog revealed many abnormalities. First of all, severe hemolytic anemia with a low number of red blood cells (RBC), low values of hematocrit (HCT) and hemoglobin (HGB), as well as severe thrombocytopenia (PLT) and leucopenia (WBC) were reported at the beginning of the infection. Towards day 4 the CBC worsened. The number of WBC increased, while the values of HCT decreased (Table 2, abnormalities are shown in bold). The biochemical parameters worsened as well. There was a significant fall in the levels of albumins, while there were slight changes in creatinine level (fall) and bilirubin concentration (increase). There were no abnormalities in levels of alanine and asparagine aminotransferases, lipase, and urea.

**Figure 1:**
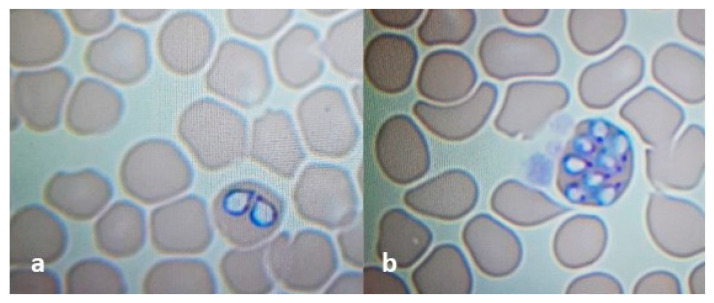
*Babesia canis* trophozoites (**a**) and merozoites (**b**) inside erythrocyte. Blood smears stained by the Diff-Quik method (100× magnifications, Nikon ECLIPSE E200).

Blood smears prepared from the EDTA-treated blood samples showed the presence of large-sized *Babesia* protozoans (Figure 1a). No morulae of *Anaplasma phagocytophilum* were detected in the analyzed blood smears.

The molecular analysis confirmed the presence of *Babesia canis* in the dogs’ blood. The sequencing analysis showed that the obtained sequence was 100% identical to the sequence of *Babesia canis* isolate 3469 (sequence ID: KX712122.1), a European *B. canis* strain belonging to the type B (*B. canis 18S* rRNA-B).

After confirmation of babesiosis (day 2), the dog was treated with imidocarb dipropionate (*Imizol,* 6.6 mg/kg, intramuscularly) initially and then again in two weeks. Despite the treatment, the dog’s clinical condition, as well as blood results, deteriorated, so supportive therapy was needed. The dog received: a blood transfusion, intravenous fluids, and an oral antibiotic (doxycycline 200 mg per day for 14 days). Ten days after admission, health status and all blood parameters improved.

### 3.2. Dog 2-Clinical Examination and Molecular Analysis

In October 2021, a 5-years old female Labrador Retriever from Zawiercie (Silesia, Poland) was presented to the veterinary clinic for dyspepsia manifestations. During the clinical examination, other unspecific symptoms were noted, such as malaise, lethargy, and weakness (Table 1). A detailed anamnesis brought the information that the dog has not left the Silesian Voivodeship since it was born, had no anti-tick prophylaxis, and it has had recent tick bites (presence of skin lesions after tick bites).

The CBC showed no abnormalities except severe thrombocytopenia (PLT) (Table 3). The biochemical analysis showed mild hyperglycemia, which may be an effect of a stress induction during a visit to the veterinary clinic, as well as a slightly lowered level of total proteins. Other biochemical parameters (alanine and asparagine aminotransferases, lipase, creatinine, and urea) were normal.

Blood smears showed the presence of pear-shaped trophozoites and merozoites of *Babesia* spp. inside red blood cells (Figure 1b).

Like in the first case, the molecular analysis confirmed the presence of *Babesia canis* in the blood of the dog. The sequencing analysis showed that the obtained sequence was 100% identical to the sequence of *B. canis* isolate 3469 (sequence ID: KX712122.1).

After confirmation of babesiosis (day 2), the dog was treated with imidocarb dipropionate (*Imizol*, 6.6 mg/kg, intramuscularly) initially and then again in two weeks. This dog also received supportive therapy (intravenous fluids and oral antibiotic-doxycycline 400 mg per day for 14 days). Seven days after admission, health status and all blood parameters improved (Table 3).

### 3.3. Ticks-Identification and Molecular Detection of Pathogens

**Figure 2:**
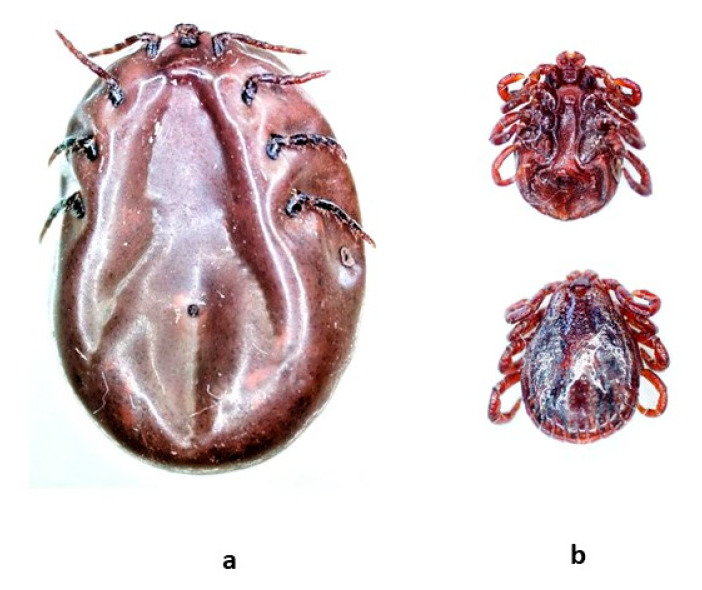
Engorged *Dermacentor reticulatus* ticks collected from “Dog 1” (stereomicroscope Leica, M205 C, camera Leica DFC 495): (**a**) female, ventral side; (**b**) male, ventral and dorsal side.

Two ticks were removed from Dog 1 at the veterinary clinic in Zawiercie, Poland. Based on their morphological traits, both ticks were identified as adult ticks (one female and one male) of the species *Dermacentor reticulatus.* Both ticks were fully engorged (Figure 2). None of the *D. reticulatus* ticks was positive for *Babesia* spp., *A. phagocytophilum*, *Borrelia burgdorferi* sensu lato, *Rickettsia* spp., or *Bartonella* spp.

## 4. Discussion

In the last years, the number of canine babesiosis incidents in Poland and in other Central European countries has increased [6,7,12,14]. This situation is probably a result of the rapid development of tourism with companion animals, such as domestic dogs and cats, and the wider spreading of ticks and *B. canis* vectors to new locations [33,34]. Furthermore, the use of sensitive detection techniques, such as PCR-based diagnostic tools, has contributed to a higher detection rate of this parasite in veterinary clinics [19,35].

So far, there have been many publications concerning the epidemiology of tick-borne diseases in domestic dogs in Europe [6,7,8,9,10,11,12,13,14,15,16,17,18,19,20,21,22,23,24,25,26,27,28,29,30,31,32,33,34,35,36,37,38,39,40,41], as well as the prevalence of tick-borne pathogens in ticks collected from pets in this area [42,43,44]. Nevertheless, the current study adds interesting new data to this subject. It is the first one to report *B. canis* infection in dogs with acute canine babesiosis symptoms from a new location in Poland, which was considered *B. canis*-free until now. Moreover, this study also presents *D. reticulatus* ticks feeding on one of the two *Babesia*-infected dogs, a dog with no travel history in the last 8 weeks before the first visit to a veterinary clinic.

In Poland, canine babesiosis is an endemic disease, which is mainly diagnosed in regions located to the east of the Vistula River (Lubelskie, Podlaskie, and Masovian Voivodships) [6,45]. Moreover, there are single reports regarding the presence of this disease in animals from western voivodships, which may be a natural consequence of the expansion of the main vector of *B. canis-D. reticulatus* in these areas [22,46,47,48]. Our study presents two cases of *B. canis* in dogs from the Silesia Voivodeship, a region of Poland, where *B. canis* was not previously detected, except for one case in the city of Żywiec, where a *Babesia*-positive dog that originally came from the vicinity of Warsaw was diagnosed [49].

Both examined dogs were thoroughly diagnosed, according to the applicable diagnostic guidelines for veterinarians, based on the clinical examinations, as well as microscopic and molecular analyses, which confirmed the presence of *B. canis* infection. In both cases, the *Babesia* infected dogs had symptoms of acute canine babesiosis with severe thrombocytopenia, elevated temperature, weakness, anemia, hemoglobinuria, vomiting, anorexia, apathy, and lethargy, which are typical symptoms of the infection caused by European *B. canis* strain belonging to the type B (*B. canis 18S* rRNA-B) [50,51,52]. In the current study, both *B. canis* sequences identified in the blood of the dogs were identical to the sequence of the more virulent type B, which was previously noted in symptomatic dogs from endemic regions of Poland, as well as other countries in Central Europe [7,16,35]. Due to the lack of information on previously reported symptomatic cases of canine babesiosis in the Silesian Voivodeship in Poland, the presence of clinical manifestations in two dogs caused the launch of other potential diagnoses (intoxication or neoplastic diseases), but no other diseases were confirmed. Detailed anamnesis showed the information that Dog 1 suffered from babesiosis in the past. There is no information about the circumstances of the previous *Babesia* infection and the travel history of this dog because it became a patient of our veterinary clinic at the beginning of 2021. Therefore, we could not exclude that the owners changed their place of residence from the area in Poland, where canine babesiosis is endemic. On the other hand, we cannot be confident that the previous diagnosis was correct because we have no information about the diagnostic procedures that were used. The studies conducted by Adaszek et al. 2011 [6] and Neelawala et al. 2021 [53] confirmed that dogs with recurrent babesiosis are noted to be prone to develop systemic complications. The case of Dog 1 was severe, with blood transfusion and strongly expressed symptoms. Therefore, relapsing babesiosis should not be ruled out. The obtained results indicate that infections with *B. canis* in dogs from the Silesian Voivodeship should be taken into account during differential diagnosis of tick-borne diseases.

In Central Europe, canine babesiosis is mostly caused by *B. canis*, and as we previously mentioned, its prevalence is strictly dependent on the presence of *D. reticulatus* ticks in the environment [54]. This tick species prefers rather humid habitats, which may positively correspond with the location of Zawiercie (50°29′15″ N, 19°24′59″ E), in the basin of the Warta and Odra rivers, among the deciduous and coniferous forests. So far, only one study described the presence of three *D. reticulatus* ticks collected from domestic dogs in two cities of Silesian Voivodeship (Racibórz and Żywiec), where the ticks from Racibórz were detected on a residential dog [49]. In order to estimate the local risk of *B. canis* infection, the prevalence rate should be determined by questing *D. reticulatus* ticks from the Silesian Voivodeship, although this species has not been recorded in the environment at this location yet.

In our study, both of the collected *D. reticulatus* ticks from Dog 1 with canine babesiosis were *Babesia* spp. negative. This result confirms that the infection status of the dog may not necessarily reflect the infection rate of ticks [20]. The role of *Ixodes ricinus*, the most common tick species in Poland, as a vector for *B. canis* should not be excluded, especially in the case of *B. canis* type A (*B. canis 18S* rRNA-A), which is responsible for a milder course of the canine babesiosis in domestic dogs [7,16,55,56]. There are studies from northern Poland [57], as well as from Croatia, Slovakia, and the Czech Republic [58,59], which show the presence of *B. canis* DNA in this tick species. *I. ricinus* is involved in the transmission of a large variety of pathogens of medical and veterinary importance, but its role in transmitting this haemoprotozoan species is unclear and needs to be further investigated.

Our study shows the occurrence of acute symptomatic canine babesiosis in dogs from the Silesian Voivodeship in Poland. These are the first reported cases of *B. canis* infections in dogs from this location, where *D. reticulatus* ticks have never been found in their natural habitat, apart from the host. Our findings should raise awareness of *B. canis* infection among dog owners and veterinarians in this region and ensure that adequate diagnostic methods are available.

## Figures and Tables

**Table 1:** “Dog 1” and “Dog 2”-a compilation of clinical symptoms.

Symptoms	Dog 1	Dog 2
Presence of ticks	X	X
Fever	X	
Lethargy	X	X
Weakness	X	X
Dehydration	X	
Anorexia	X	X
Splenomegaly	X	
Hemoglobinuria	X	
Malaise	X	X
Fatigue	X	
Pale mucous membranes	X	
Vomiting	X	
Diarrhea	X	X

**Table 2:** “Dog 1”-a compilation of the complete blood count (CBC) parameters.

Parameters CBC	Day 1	Day 2	Day 4	Day 6	Day 7	Day 10	Reference Range
WBC [10^9^/L]	**3.09**	**3.42**	**27.94**	**40.62**	**50.21**	**23.37**	6.00–17.00
LYM [10^9^/L]	**0.45**	**0.57**	5.14	2.98	No data	3.34	0.80–5.30
MONO [10^9^/L]	0.19	0.29	**2.88**	**2.20**	No data	**2.17**	0.00–1.50
NEU [10^9^/L]	2.40	2.33	**19.08**	**17.50**	No data	**17.46**	3.20–12.30
EOS [10^9^/L]	0.05	0.23	0.84	0.70	No data	0.40	0.00–1.50
HCT [%]	48.7	**32.4**	**10.9**	**16.5**	**17.4**	33.7	32.5–58.00
RBC [10^12^/L]	7.54	**4.94**	**1.46**	**2.3**	**2.32**	**3.86**	5.10–8.50
HGB [g/dL]	17.6	11.5	**5.1**	**6.7**	**6.9**	**10.5**	11.00–19.5
PLT [10^9^/L]	**30.00**	**13.00**	**36.00**	**110**	165	338	117.00–490.00
MCV [fL]	64.6	65.6	74.4	71.7	74.9	**87.1**	60.00–76.00
MCHC [g/dL]	36.2	35.5	**47.00**	**40.3**	**39.8**	31.3	30.00–38.00
MCH [pg]	23.4	23.3	**34.9**	**28.9**	**29.8**	**27.3**	20.00–27.00

Explanations: WBC, white blood cells; LYM, lymphocytes; MONO, monocytes; NEU, neutrophils; EOS, eosinophils; HCT, hematocrit; RBC, red blood cells; HGB, hemoglobin; PLT, platelets; MCV, mean corpuscular volume; MCHC, mean corpuscular hemoglobin concentration; MCH, mean cell hemoglobin.

**Table 3:** “Dog 2”-a compilation of the complete blood count (CBC) parameters.

Parameters CBC	Day 1	Day 4	Day 9	Reference Range
WBC [10^9^/L]	6.55	**19.55**	12.37	6.00–17.00
LYM [10^9^/L]	No data	No data	No data	0.80–5.30
MONO [10^9^/L]	No data	No data	No data	0.00–1.50
NEU [10^9^/L]	No data	No data	No data	3.20–12.30
EOS [10^9^/L]	0.2	**2.1**	0.67	0.00–1.50
HCT [%]	34.8	40.2	44.9	32.5–58.00
RBC [10^12^/L]	5.34	6.04	6.69	5.10–8.50
HGB [g/dL]	12.4	14.1	15.7	11.00–19.5
PLT [10^9^/L]	**25.00**	**67.00**	376.00	117.00–490.00
MCV [fL]	65.2	66.6	67.00	60.00–76.00
MCHC [g/dL]	35.6	35.1	35.00	30.00–38.00
MCH [pg]	23.2	23.4	23.4	20.00–27.00

Explanations: WBC, white blood cells; LYM, lymphocytes; MONO, monocytes; NEU, neutrophils; EOS, eosinophils; HCT, hematocrit; RBC, red blood cells; HGB, hemoglobin; PLT, platelets; MCV, mean corpuscular volume; MCHC, mean corpuscular hemoglobin concentration; MCH, mean cell hemoglobin.

## Data Availability

Not applicable.
